# Lipid phosphate phosphatase inhibitors locally amplify lysophosphatidic acid LPA_1_ receptor signalling in rat brain cryosections without affecting global LPA degradation

**DOI:** 10.1186/1471-2210-12-7

**Published:** 2012-06-11

**Authors:** Niina Aaltonen, Marko Lehtonen, Katri Varonen, Gemma Arrufat Goterris, Jarmo T Laitinen

**Affiliations:** 1 School of Pharmacy, University of Eastern Finland, P.O. Box 1627, 70211, Kuopio, Finland; 2 School of Medicine, Institute of Biomedicine/Physiology, University of Eastern Finland, P.O. Box 1627, 70211, Kuopio, Finland

## Abstract

**Background:**

Lysophosphatidic acid (LPA) is a signalling phospholipid with multiple biological functions, mainly mediated through specific G protein-coupled receptors. Aberrant LPA signalling is being increasingly implicated in the pathology of common human diseases, such as arteriosclerosis and cancer. The lifetime of the signalling pool of LPA is controlled by the equilibrium between synthesizing and degradative enzymatic activity. In the current study, we have characterized these enzymatic pathways in rat brain by pharmacologically manipulating the enzymatic machinery required for LPA degradation.

**Results:**

In rat brain cryosections, the lifetime of bioactive LPA was found to be controlled by Mg^2+^-independent, N-ethylmaleimide-insensitive phosphatase activity, attributed to lipid phosphate phosphatases (LPPs). Pharmacological inhibition of this LPP activity amplified LPA_1_ receptor signalling, as revealed using functional autoradiography. Although two LPP inhibitors, sodium orthovanadate and propranolol, locally amplified receptor responses, they did not affect global brain LPA phosphatase activity (also attributed to Mg^2+^-independent, N-ethylmaleimide-insensitive phosphatases), as confirmed by P_i_ determination and by LC/MS/MS. Interestingly, the phosphate analog, aluminium fluoride (AlF_x_^-^) not only irreversibly inhibited LPP activity thereby potentiating LPA_1_ receptor responses, but also totally prevented LPA degradation, however this latter effect was not essential in order to observe AlF_x_^-^-dependent potentiation of receptor signalling.

**Conclusions:**

We conclude that vanadate- and propranolol-sensitive LPP activity locally guards the signalling pool of LPA whereas the majority of brain LPA phosphatase activity is attributed to LPP-like enzymatic activity which, like LPP activity, is sensitive to AlF_x_^-^ but resistant to the LPP inhibitors, vanadate and propranolol.

## Background

Lysophosphatidic acid (LPA, 1- or 2-acyl-*sn*-glycero-3-phosphate) is a signalling phospholipid mediating multiple biological responses, such as cellular proliferation, prevention of apoptosis, and platelet aggregation, and is involved in the development and function of the nervous, cardiovascular, immune, and reproductive systems [1,2]. Aberrant LPA signalling has been claimed to be associated with the pathology of common human diseases, such as arteriosclerosis [3] and cancer [4]. Signalling by LPA is mainly mediated through specific G protein-coupled receptors (GPCRs) [5-7]. The receptors for LPA are widely expressed, being found in the brain, circulation and digestive tract. Currently, there are five GPCRs that have been identified as bona fide receptors for LPA (LPA_1-5_) along with a putative sixth receptor (LPA_6_) [8].

Physiologically relevant levels of LPA can be found in serum and other body fluids. In addition, several cell types, including platelets, adipocytes, and ovarian cancer cells, can produce and release LPA. Significant amounts of LPA have been detected from brain tissue [9-11]. It is postulated that the majority of bioactive LPA is generated extracellularly from lysophospholipids, such as lysophosphatidylcholine (LPC), by the plasma ecto-enzyme, lysophospholipase D, identical to autotaxin, an autocrine motility factor originally isolated from the conditioned medium of cancer cells [12]. Intracellularly, LPA can be generated by phospholipase A_1_/A_2_ (PLA_1_/PLA_2_, respectively) -catalyzed deacylation of phosphatidic acid (PA) [13,14]. Other proposed pathways for LPA generation include de novo biosynthesis either from glycerol-3-phosphate (GP) by glycerol-3-phosphate acyltransferase or from monoacylglycerol (MAG) by monoacylglycerol kinase [15].

**Figure 1:**
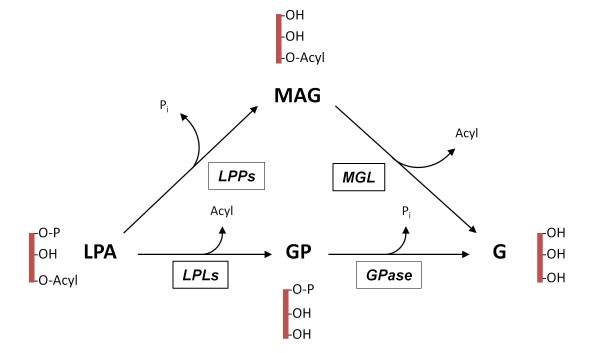
**The two enzymatic pathways generating inorganic phosphate (P**_**i**_**) as a result of LPA degradation.** In the LPA → MAG → G pathway, lipid phosphate phosphatases (LPPs) dephosphorylate LPA resulting in the generation of equimolar amounts of monoacylglycerol (MAG) and P_i_. MAG is further hydrolyzed by monoglyceride lipase (MGL), resulting in cleavage of the acyl moiety and the formation of glycerol (G). In the LPA → GP → G pathway, lysophospholipases (LPLs) catalyze the deacylation of LPA with the concomitant formation of glycerophosphate (GP). GP can be further metabolized by GP phosphatase (GPase) activity thereby generating equimolar amounts of P_i_ and G.

^35^ S]GTPγS autoradiography represents a powerful functional approach to anatomically localize receptor-dependent G_i/o_ protein activity directly in brain cryosections (for reviews, see [24,25]). In addition to a receptor’s anatomical distribution, ^35^ S]GTPγS autoradiography can monitor the receptor-G protein axis in its native cellular microenvironment and is therefore often referred to as functional autoradiography. Our previous studies have demonstrated that brain sections retain the capacity to generate endogenous GPCR agonists, such as adenosine and LPA, during incubation. This results in tonic adenosine A_1_ and LPA receptor activity in anatomically defined brain regions and therefore serves as a convenient functional readout to monitor agonist activity at the two receptors [26-28]. The LPA-evoked ^35^ S]GTPγS binding response in rat brain sections reflects LPA_1_ receptor activity, as it is sensitive to the LPA_1/3_-selective antagonist Ki16425 and is restricted to the developing white matter tracts [24,26,29]. This labelling pattern faithfully mirrors the known expression pattern of LPA_1_ receptors in the developing rat brain [30-33].

After being produced, the lifetime of the signalling pool of LPA is thought to be controlled by enzymatic degradation. LPA is rapidly dephosphorylated by a family of integral membrane proteins known as lipid phosphate phosphatases (LPPs) [16-18]. The LPP family (also known as PAP2) comprises four members (LPP_1_, LPP_2_, LPP_3_ and a splice variant LPP_1a_) which dephosphorylate their lipid substrates, namely LPA, PA, sphingosine 1-phosphate (S1P), and ceramide 1-phosphate [19-21]. All the LPP subtypes are expressed in the brain [18,19,22] but very little is known about their functional roles. A hallmark of LPP activity is that it does not require Mg^2+^ and is resistant to the alkylating agent, N-ethylmaleimide (NEM) [23]. The other known pathways for LPA metabolism include de novo formation of PA by lysophosphatidic acid acyltransferase and lysophospholipase -catalyzed hydrolysis of the acyl group to form glycerophosphate [15]. The two degradative pathways generating inorganic phosphate (P_i_) as a result of LPA degradation are depicted in Figure 1.

Our previous studies indicated that the enzymatic machinery generating and metabolizing membrane-derived lipid mediators was well preserved in brain cryosections. We recently demonstrated that a comprehensive elimination of the enzymatic hydrolysis of the endocannabinoid 2-arachidonoylglycerol (2-AG) in brain sections leads to 2-AG accumulation and subsequent cannabinoid CB_1_ receptor activation, as successfully revealed using functional autoradiography [34]. Using this approach, we show here that pharmacological inhibition of LPP activity in brain sections with vanadate or propranolol results in amplification of LPA_1_ receptor signalling with no net effect on global LPA phosphatase activity at the bulk brain level, also attributable to Mg^2+^ -independent, NEM-resistant LPP-like phosphatases. We show further that the phosphate analog AlF_x_^-^ not only potentiates LPA_1_ receptor signalling, but also totally prevents LPA degradation, resulting in the accumulation of several LPA species in brain sections, as demonstrated by LC/MS/MS measurements. The presently described approach offers a versatile tool to monitor the strength of lipid-GPCR signalling axis in anatomically defined brain structures and may prove useful also for further studies exploring enzymatic pathways estimating the lifetime of still uncharacterized endogenous signalling lipids.

## Results

### The LPP inhibitors Na_3_VO_4_ and propranolol locally amplify LPA_1_ receptor signalling without affecting global LPA degradation

**Figure 2:**
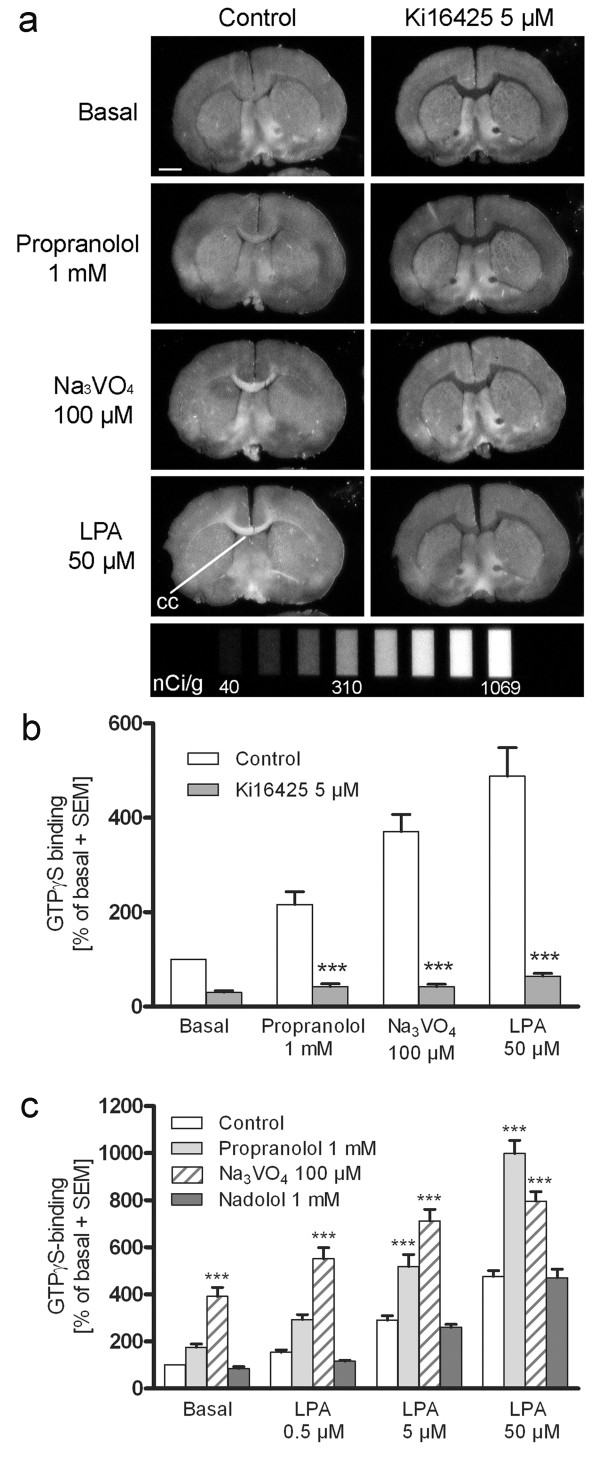
**Vanadate and propranolol evoke LPA-mimicking [**^**35**^ **S]GTPγS binding response that is sensitive to LPA**_**1/3**_**receptor antagonist.** (**a**) Coronal brain sections were incubated using a three-step autoradiography protocol as detailed in Methods. Test chemicals were included during the [^35^ S]GTPγS labelling step (step 3) in the presence of 0.1% BSA. Treatment with propranolol (1 mM) or Na_3_VO_4_ (100 μM), results in G protein activity in the LPA_1_ receptor enriched white matter tracts, a response that is mimicked by exogenous LPA (50 μM). The LPA_1/3_ receptor antagonist Ki16425 (5 μM) abolishes all the evoked responses in the white matter regions, including the tonic LPA_1_ signal observed under basal conditions (cc, corpus callosum). Scale bar = 2 mm. The eight-point [^14^ C] standard used in the quantification is shown at the bottom of image. (**b**) and (**c**) Quantitative data of the binding responses including the dose response for exogenous LPA (0.5 μM to 50 μM) with or without the simultaneous treatment with the inhibitors. Autoradiography films were digitized and [^35^ S]GTPγS binding was quantified from the corpus callosum of coronal sections of 4 week-old rat brain, as described in Methods. Quantitative data are calculated as nCi/g equivalents with non-specific binding subtracted from total binding. The data are expressed as a percentage of basal binding (mean + SEM) from six individual animals (n = 6). Significance level: (b) ***p < 0.001 compared to the treatment without Ki16425 (**c**) ***p < 0.001 compared to control in each LPA concentration.

The LPP-mediated degradation of LPA is susceptible to the phosphatase inhibitor sodium orthovanadate (Na_3_VO_4_) [35] and to propranolol [20,36], better known as a classical β-adrenoceptor blocking agent e.g. used in the treatment of hypertension. Another β-blocker, nadolol, has no demonstrable LPP inhibiting capacity [36] and therefore can serve as a useful control compound. We tested the effects of these compounds on basal LPA_1_ receptor signalling. Treatment of brain sections with propranolol (1 mM) or Na_3_VO_4_ (100 μM) resulted in stimulated ^35^ S]GTPγS binding responses that were restricted to the white matter areas of 4 week-old rat brain (Figure 2a). The observed labelling pattern was fully reproduced by the addition of exogenous LPA (50 μM) and all the evoked responses, including the tonic LPA_1_ receptor activity observed under basal conditions, were abolished by treatment with the LPA_1/3_ receptor selective antagonist Ki16425 (5 μM) (Figure 2a and b). Previously, we demonstrated that Ki16425 dose-dependently diminished the basal and LPA-evoked ^35^ S]GTPγS binding responses in the white matter tracts (IC_50_ values 35 ± 9 nM and 87 ± 19 nM, respectively) [28]. Dose–response studies revealed that the maximal effective concentrations for Na_3_VO_4_ and propranolol were 100 μM and 1 mM, respectively (See Additional file 1: The maximal effective concentrations for Na_3_VO_4_ and propranolol). As expected, treatment with nadolol (1 mM) had no effect on basal LPA_1_ receptor signalling (Figure 2c, Additional file 2: Propranolol, but not nadolol, induces LPA mimicking binding response). Both Na_3_VO_4_ and propranolol, but not nadolol, amplified the LPA-evoked (0.5 μM to 50 μM) binding responses (Figure 2c). To rule out the direct agonism of propranolol and vanadate at the LPA_1_ receptor, we performed classical filtration-based ^35^ S]GTPγS binding assay and found that neither compound was able to stimulate ^35^ S]GTPγS binding to the rat cerebellar membranes, whereas exogenous LPA evoked a dose-dependent response (See Additional file 3: Propranolol and vanadate do not activate LPA_1_ receptors). We additionally observed that the LPA_3_ receptor-preferring agonist (2 S)-OMPT (0.5 μM to 50 μM) induced only a weak response in the brain of a 4-week old rat when compared to signal achieved with exogenous LPA (10 μM) (See Additional file 4: Comparison of the ^35^ S]GTPγS binding responses between LPA and (2 S)-OMPT). This lends further support to the argument that observed LPA-evoked signalling is reflecting the activity of the myelin enriched LPA_1_ receptors instead of LPA_3_ receptors that are expressed to a lesser degree in the brain [5]. Finally, we found that exogenously added autotaxin substrate LPC did not boost tonic LPA_1_ receptor activity suggesting that tonic LPA_1_ activity in brain sections is not due to LPA formed as a result of autotaxin activity (See Additional file 5: Autotaxin is not responsible for tonic LPA_1_ activity).

**Figure 3:**
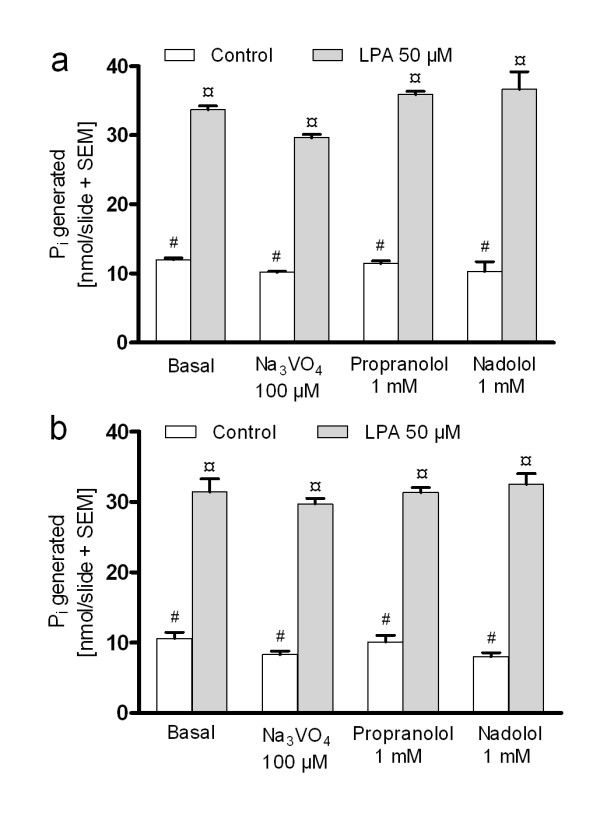
**Brain tissue dephosphorylates LPA through LPP-like phosphatases that are resistant to propranolol and Na**_**3**_**VO**_**4**_**.** Slides with three horizontal rat brain sections underwent the autoradiography mimicking incubation protocol, as detailed in Methods (**a**), or were pretreated with NEM (5 mM) followed by the autoradiography mimicking protocol with the exception that Mg^2+^ was omitted from the incubation buffer (**b**). Following 90 min incubation in the presence of 0.1% BSA together with the indicated combinations and concentrations of the compounds, the assay buffer was quantitatively collected and the P_i_ content was determined as described in Methods. Note that the bulk of LPA degradation is due to NEM resistant and Mg^2+^ independent phosphatase activity (compare a and b) and that Na_3_VO_4_ (100 μM), propranolol (1 mM) or nadolol (1 mM) do not affect basal or LPA-derived P_i_ generation in a statistically significant manner. The data are expressed as nmol P_i_ generated per slide (mean + SEM) and were obtained from three independent experiments performed in triplicate (n = 3). Significance level: Bars indicated with ¤ represent significant difference (*p* < 0.001) to bars indicated with # but there is no significant difference between the bars marked with the same symbol.

The LPPs catalyze the hydrolysis of the phosphate group of their lipid substrates resulting in the generation of inorganic phosphate (P_i_). The measurement of the released P_i_ offers a straightforward way to monitor LPP activity [37]. In preliminary experiments, incubation of brain sections with exogenously added LPA, PA and S1P resulted in P_i_ formation, indicating that lysophospholipid-degrading phosphatases were active under the assay conditions employed (See Additional file 6: Phosphate generation from exogenous LPA, PA, and S1P). To confirm that P_i_ generation was dependent on brain tissue, empty slides were incubated under identical conditions but in this case, there was no generation of P_i_ from LPA (data not shown). Further studies with LPA (50 μM) indicated, that LPP-like phosphatase activity accounted for the majority of LPA degradation, as ~ 93% of LPA-derived P_i_ was formed as a result of Mg^2+^-independent, NEM-resistant phosphatase activity (Figure 3). In the routine assay buffer containing Mg^2+^, 46 ± 1% (mean ± SEM, n = 3) of exogenous LPA (50 μM, corresponding to 47.5 nmol potentially available P_i_ per slide) was degraded during the 90 min incubation whereas in the Mg^2+^-free assay buffer supplemented with NEM (5 mM), the respective figure was 44 ± 3%. However, neither the LPP inhibitors Na_3_VO_4_ (100 μM), propranolol (1 mM), nor nadolol (1 mM) affected total LPA phosphatase activity in a statistically significant manner, assessed at the bulk level of brain sections (Figure 3). In line with this, when the LPA content of brain sections treated with Na_3_VO_4_ or propranolol was analyzed using LC/MS/MS, there was no significant accumulation of LPA when compared to control sections that were incubated in the absence of these inhibitors (data not shown). We undertook a search for additional inhibitors in an attempt to identify compounds that could comprehensively target the global pool of LPA phosphatases in brain sections.

### The phosphate analog aluminium fluoride amplifies LPA_1_ receptor signalling and totally prevents LPA degradation resulting in bulk accumulation of endogenous LPA species

**Figure 4:**
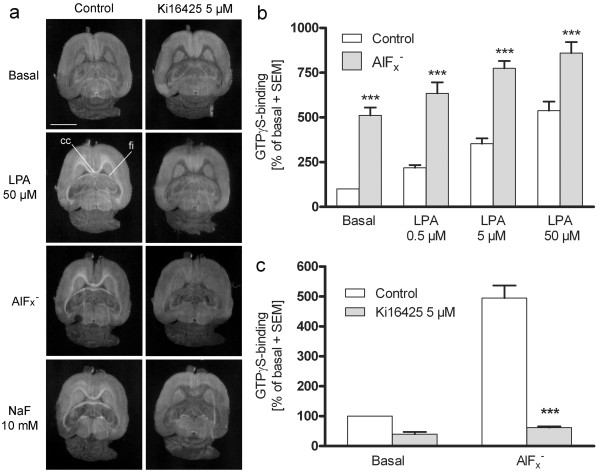
**AlF**_**x**_^**-**^**and NaF evoke LPA-mimicking [**^**35**^ **S]GTPγS binding response that is sensitive to LPA**_**1/3**_**receptor antagonist.** (**a**) Horizontal brain sections were incubated using a three-step autoradiography protocol as described in Methods. AlF_x_^-^ (NaF 10 mM + AlCl_3_ 50 μM) was included during the 40 min preincubation step (step 1), NaF (10 mM) was included in step 2, and LPA (50 μM) in step 3, whereas Ki16425 (5 μM) was present throughout all steps. The buffer in all the steps additionally contained 0.1% BSA. Treatment with AlF_x_^-^ and NaF results in region-specific G protein activity that is restricted to the LPA_1_ receptor enriched white matter tracts, a response that is mimicked by exogenous LPA and is sensitive to Ki16425 (cc, corpus callosum; fi, fimbria of the hippocampus). Scale bar = 5 mm. (**b**) and (**c**) Quantitative data of the binding responses. Autoradiography films were digitized and [^35^ S]GTPγS binding was quantified from corpus callosum of the coronal sections of 4 week-old rat brain, as detailed in Methods. Data are calculated as nCi/g equivalents with non-specific binding subtracted from the total and are expressed as a percentage of basal binding (mean + SEM) from six individual animals (n = 6). Significance level: (**b**) ***p < 0.001 compared to control in each LPA concentration (**c**) ***p < 0.001 compared to the treatment without Ki16425.

Sodium fluoride (NaF) is commonly used as a phosphatase inhibitor. Aluminium fluoride (AlF_x_^-^) acts as a transition stage phosphate analog also capable of inhibiting several phosphatases [38]. AlF_x_^-^ forms spontaneously in aqueous solutions in the presence of aluminium (Al^3+^) and fluoride ions. We pretreated brain sections with NaF or AlF_x_^-^ in order to test whether these compounds could affect LPA receptor activity and/or LPA degradation. Interestingly, when sections were pretreated with AlF_x_^-^ (AlCl_3_ 50 μM + NaF 10 mM), a stimulated ^35^ S]GTPγS binding response throughout the LPA_1_ receptor-enriched white matter regions was evident (Figure 4a and b). This labelling pattern was fully mimicked by the addition of exogenous LPA (0.5 μM to 50 μM) (Figure 4a and b), and was blocked by Ki16425 (5 μM) (Figure 4a and c). Similarly, pretreatment of brain sections with NaF (10 mM) resulted in stimulated ^35^ S]GTPγS binding responses throughout the white matter regions (Figure 4a). Pretreatment with these compounds was sufficient to evoke LPA_1_ receptor signalling, suggesting that in contrast to the reversibly acting inhibitors Na_3_VO_4_ and propranolol (that needed to be present during the ^35^ S]GTPγS labelling step), AlF_x_^-^ and NaF had inhibited LPP activity in an irreversible manner.

**Figure 5:**
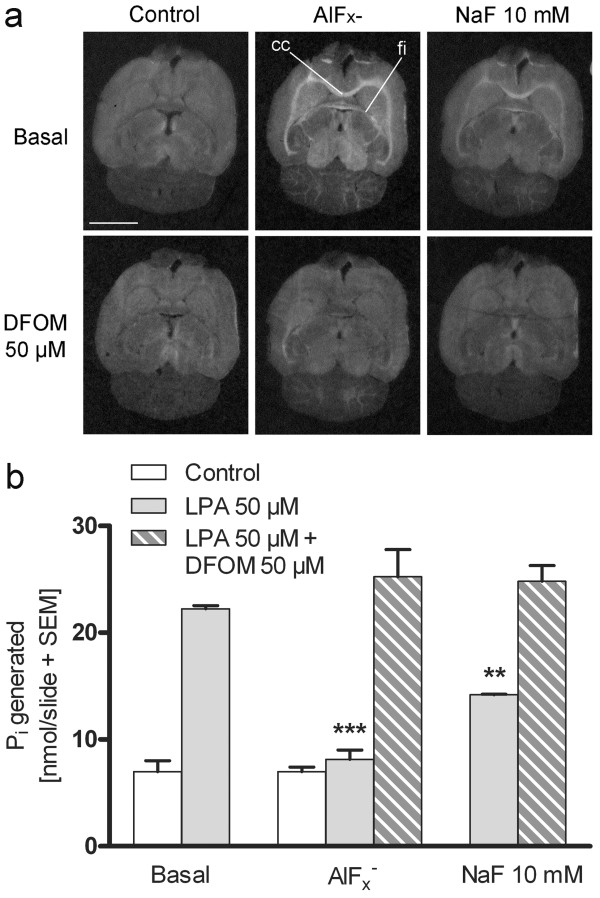
**AlF**_**x**_^**-**^**(not NaF) generates the LPA**_**1**_**receptor-mediated response; AlF**_**x**_^**-**^**also blocks the LPA phosphatase activity.** (**a**) Functional autoradiography using horizontal brain sections was performed using a three-step protocol as detailed in Methods. AlF_x_^-^ (NaF 10 mM + AlCl_3_ 50 μM) was included during the 40 min pre-incubation step (step 1), NaF (10 mM) was present in step 2 whereas deferoxamine mesylate (DFOM, 50 μM) was present throughout steps 1–3 and in addition during a 10 min pre-incubation prior to AlF_x_^-^ treatment. The buffer in step 3 additionally contained 0.1% BSA. Both NaF and AlF_x_^-^ induce [^35^ S]GTPγS binding in the LPA_1_ receptor enriched white matter areas. The responses to AlF_x_^-^ and NaF are totally abolished with the aluminium and iron(III) chelator DFOM (cc, corpus callosum; fi, fimbria of the hippocampus). Scale bar = 5 mm. (**b**) Slides with two horizontal brain sections underwent the three-step autoradiography mimicking protocol as detailed in Methods. AlF_x_^-^ (NaF 10 mM + AlCl_3_ 50 μM) and NaF (10 mM) were present during the 90 min incubation step (step 3). DFOM (50 μM) was present in steps 2 and 3. The buffer in step 3 additionally contained 0.1% BSA. After the final incubation step, the assay buffer was quantitatively collected and the P_i_ content was determined as described in Methods. Note that NaF partially and AlF_x_^-^ totally inhibit LPA-derived P_i_ formation and that DFOM reverses these actions. The data are expressed as nmol P_i_ generated per slide (mean + SEM) and are derived from three independent experiments performed in triplicate (n = 3). Significance level: ***p < 0.001 and **p < 0.01 compared to the treatment with LPA alone.

We wished to explore in more detail the mode of inhibition of these compounds, as well as the behaviour of NaF in our experimental setting. This was justified by the fact that aluminium is a common constituent of glassware and F^-^ can etch it from the glass. Deforoxamine mesylate (DFOM) is an aluminium and iron(III) chelator that can be used in experimental settings to reveal if aluminium is present in the system. When brain sections were treated with DFOM (50 μM), the responses to AlF_x_^-^ and NaF (10 mM) in functional autoradiography were totally abolished (Figure 5a). It is noteworthy that DFOM needed to be added together with AlF_x_^-^ or NaF in order to achieve this reversal; if added after pretreatment with AlF_x_^-^ or NaF, DFOM was ineffective (data not shown). These studies indicate that AlF_x_^-^ acted as an irreversible inhibitor of brain LPP activity thereby amplifying tonic LPA_1_ receptor activity. The general phosphatase inhibitor, NaF per se, did not inhibit LPPs, but mimicked the action of AlF_x_^-^ based on the ability of F^-^ to etch Al^3+^ from the glass slides.

We were curious to examine whether AlF_x_^-^ could also inhibit LPA degradation at the bulk brain level. When sections were pretreated with AlF_x_^-^ but then omitted from all subsequent steps, AlF_x_^-^ readily facilitated LPA_1_ receptor signalling (Figure 4, Figure 5a), but such a pretreatment did not inhibit LPA degradation in a statistically significant manner. Degradation of exogenous LPA (50 μM) alone yielded 22.6 ± 1.2 nmol P_i_ per slide whereas pretreatment with AlF_x_^-^ followed by incubation with exogenous LPA yielded 21.4 ± 0.4 nmol P_i_ per slide (mean ± SEM, n = 3). However, when added together with LPA, AlF_x_^-^ totally (and NaF partially) blocked the formation of LPA-derived P_i_, thus providing evidence of the ability of these compounds to inhibit the vanadate- and propranolol-insensitive pool of LPA phosphatases in a reversible manner (Figure 5b). Treatment with DFOM (50 μM) totally prevented the ability of AlF_x_^-^ and NaF to inhibit the degradation of LPA (Figure 5b), indicating that AlF_x_^-^, rather than NaF, was the active compound.

**Figure 6:**
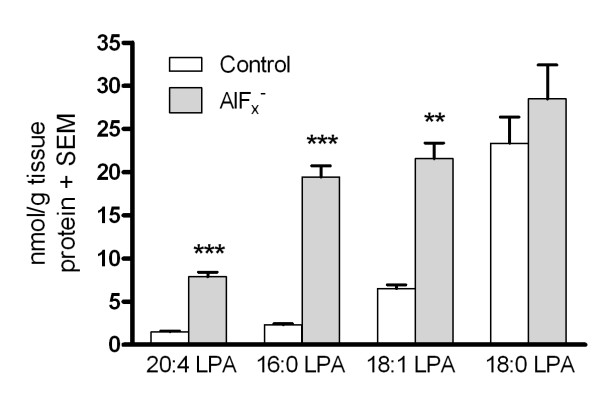
**Treatment with AlF**_**x**_^**-**^**results in accumulation of several endogenous LPA species in rat brain sections.** The slides with two horizontal brain sections were treated with AlF_x_^-^ (NaF 10 mM + AlCl_3_ 50 μM) for 40 min followed by extraction of LPA species, as described in Methods. The samples were analyzed with LC/MS/MS for four endogenous LPA species, 16:0 LPA, 18:0 LPA, 18:1 LPA and 20:4 LPA. The amounts of three of these LPA species are increased in a statistically significant manner in AlF_x_^-^ treated sections as compared to control sections. The results are expressed as nmol/g of tissue protein (mean + SEM) and are derived from three individual experiments performed in triplicate (n = 3). Significance level: ***p < 0.001 and **p < 0.01 compared to control.

To further explore the consequences of total inhibition of LPA phosphatase activity, AlF_x_^-^ -treated brain sections were incubated for 40 min in autoradiography buffer, the buffer was removed and tissue LPA content extracted using chloroform-methanol, followed by LC/MS/MS analysis. Four LPA species with different acyl substitutions (16:0 LPA, 18:1 LPA, 18:0 LPA and 20:4 LPA) were examined in the present study. The relative abundances of the four LPA species in brain sections incubated under control conditions were 20:4 LPA ≈ 16:0 LPA < 18:1 LPA < 18:0 LPA. The amounts of three of these species (16:0 LPA, 18:1 LPA and 20:4 LPA) were significantly increased after AlF_x_^-^ treatment when compared to control sections (Figure 6). These experiments indicate that total blockade of LPA phosphatase activity with AlF_x_^-^ treatment resulted in accumulation of several endogenous LPA species at the bulk brain level. However, no such bulk LPA accumulation was required to observe the AlF_x_^-^ -evoked potentiation of LPA_1_ receptor signalling.

### The LPA → MAG → G pathway efficiently degrades exogenous LPA whereas the LPA → GP → G pathway is inactive

**Figure 7:**
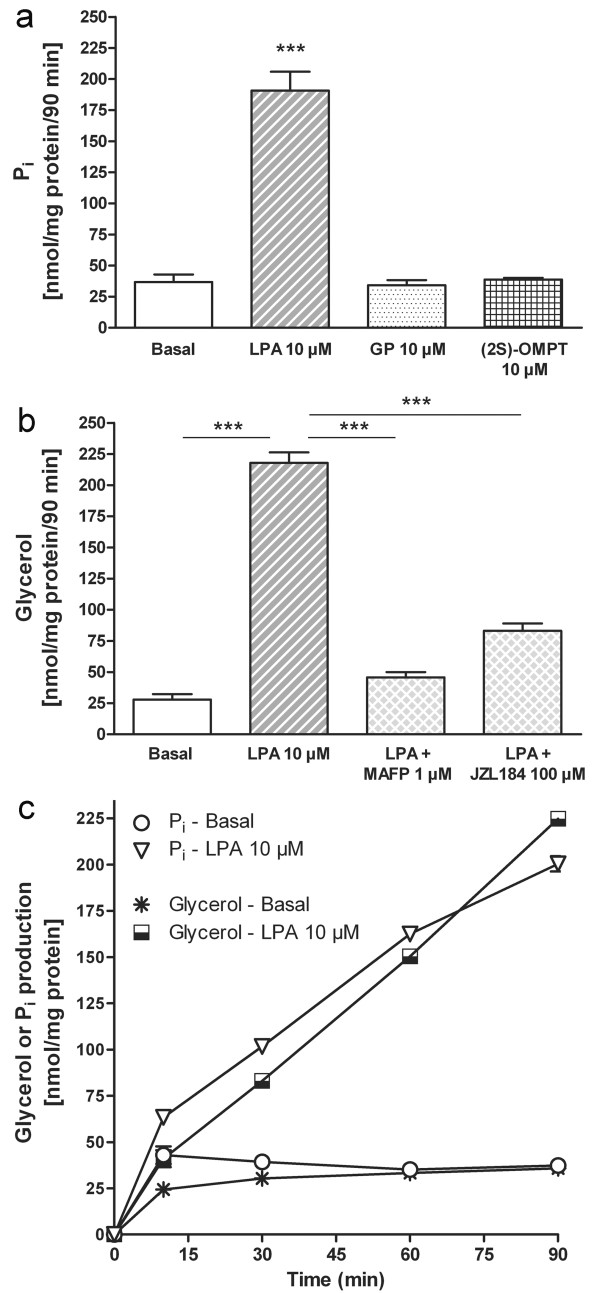
**Rat cerebellar membranes principally metabolize LPA via the LPA → MAG → G pathway.** (**a**) Rat cerebellar membranes (1 μg/well) were incubated in the absence (basal) or presence of LPA, glycerol 3-phosphate (GP), or the phosphatase-resistant LPA analog (2 S)-OMPT (10 μM final concentration each). P_i_ generation was kinetically monitored for 90 min using a fluorescent assay as described in Methods. Rat cerebellar membranes generate P_i_ from exogenous LPA but not from exogenous GP or (2 S)-OMPT. The data are expressed as nmol of P_i_ generated per mg of protein during the 90 min incubation (mean + SEM, n = 4 for basal and LPA, n = 3 for GP and (2 S)-OMPT). Significance level: ***p < 0.001 compared to basal. (**b**) Rat cerebellar membranes were pretreated for 30 min with DMSO or the broadly-acting serine hydrolase inhibitor MAFP (1 μM) or the MGL-specific inhibitor JZL184 (100 μM). This was followed by 90 min incubation in the absence (basal) or presence of LPA (10 μM final concentration). Glycerol generation was kinetically monitored for 90 min using a fluorescent assay as described in Methods. Rat cerebellar membranes readily generate glycerol from exogenous LPA; this response is largely blocked by MAFP and partially so by JZL184. The data are expressed as nmol of glycerol generated per mg of protein during the 90 min incubation (mean + SEM, n = 4). Significance level: ***p < 0.001 compared to either basal or particular treatment. (**c**) Simultaneous kinetic monitoring of P_i_ and glycerol generation from exogenous LPA by rat cerebellar membranes during 90 min incubation. Note that P_i_ generation precedes and exceeds that of glycerol during the early time-points (10–30 min) suggesting temporal first phosphatase (LPA → MAG) then lipase (MAG → G) action. The data are expressed as nmol of P_i_ or glycerol generated per mg of protein and are means of duplicate wells from one representative experiment.

In addition to the LPA → MAG → G pathway (Figure 1), another P_i_ generating pathway for LPA degradation involves its deacylation to form glycerophosphate which is further dephosphorylated by glycerophosphatases to glycerol (the LPA → GP → G pathway) (Figure 1). Since there are two degradative pathways for LPA that potentially release P_i_, we wished to clarify which pathway accounts for LPA degradation in our model. Rat cerebellar membranes have been extensively used in our laboratory to study degradation of labile lipid messengers such as endocannabinoids [34,39]. Since LPA_1_ receptors are known to be present in the cerebellum [30] we performed kinetic monitoring of P_i_ production from exogenous LPA and glycerol 3-phosphate (GP) after incubation with rat cerebellar membranes in 96-well-format. In this setting, cerebellar membranes generated P_i_ from LPA (10 μM) but there was no P_i_ generation from GP (10 μM) (Figure 7a), indicating that the P_i_ formed from LPA in our experimental setting is principally due to LPP/LPP-like activity. As a further proof, we used the dephosphorylation-resistant thio-analog of LPA, (2 S)-OMPT, that is also the LPA_3_ receptor_._-preferring agonist. As expected, there was no P_i_ generation from (2 S)-OMPT (10 μM) (Figure 7a).

Since both of the two P_i_ releasing pathways for LPA degradation finally produce glycerol, we assessed cerebellar membrane-dependent glycerol generation from exogenous LPA in brain tissue. Monoglyceride lipase (MGL) is believed to be mainly responsible for the MAG → G conversion. In addition, two novel α/β-hydrolase domain containing proteins, ABHD6 and ABHD12, have been identified to hydrolyze brain endocannabinoid 2-arachidonoylglycerol (2-AG) [40] and together the three serine hydrolases account for ~99% of brain 2-AG hydrolase activity [41]. It is therefore likely that in addition to MGL, ABHD6 and ABHD12 are involved in the degradation of both 1- and 2-monoacylglycerols. To delineate the relative contributions of the three hydrolases, we pretreated cerebellar membranes with two serine hydrolase inhibitors, methylarachidonoylfluorophosphonate (MAFP) and compound JZL184. The former is a potent, non-selective inhibitor of MGL [39,42] that also inhibits ABHD6/ABHD12, whereas the latter is a MGL-selective inhibitor [43]. As expected, in rat cerebellar membranes incubated together with LPA (10 μM), glycerol production closely matches with P_i_ generation (Figure 7a and b), indicating that MAG → G conversion takes place under the assay conditions employed. With MAFP pretreatment (1 μM), LPA-derived glycerol production was decreased by 91% (Figure 7b). With JZL184 pretreatment (100 μM), the corresponding reduction was 71% (Figure 7b). The selectivity of the inhibitors towards MGL likely explains the difference in the inhibition of glycerol production from LPA between the two inhibitors. The time-dependent generation of P_i_ and glycerol from exogenous LPA is presented in Figure 7c. It appears that P_i_ generation precedes that of G, a finding supporting sequential actions of phosphatases and lipases on the LPA → MAG → G pathway.

## Discussion

Functional autoradiography provides a straightforward approach to study the proximal step of signalling of various G_i/o_-coupled receptors in brain cryosections. We recently demonstrated that brain sections retain the capacity to generate endocannabinoids during incubations i.e. evidence of the ability of the brain sections to preserve sufficient functional enzymatic machinery to generate endogenous GPCR activating ligands [34]. The lifetime of the signalling pool of LPA is thought to be controlled by the equilibrium between synthesizing and degradative enzymatic activity. In the current study, we have characterized these enzymatic pathways and their role in tonic LPA_1_ receptor activity by pharmacologically manipulating the enzymatic machinery required for LPA degradation. We observed that in brain sections, the lifetime of bioactive LPA is controlled by Mg^2+^-independent, NEM-insensitive phosphatase activity attributable to LPPs. Pharmacological inhibition of this LPP activity by AlF_x_^-^, propranolol or sodium orthovanadate amplified LPA_1_ receptor signalling, as revealed using functional autoradiography. We provided further evidence to show that the majority of brain LPA phosphatase activity seems to be carried out by LPP-like enzymatic activity which like LPP activity, is sensitive to AlF_x_^-^ but appears to be resistant to the two other LPP inhibitors, vanadate and propranolol. Finally, we demonstrated that degradation of exogenous LPA is almost entirely channelled via the LPA → MAG → G pathway and that MGL accounts for the majority of oleylglycerol-hydrolyzing activity in brain tissue.

All the three subtypes of Mg^2+^-independent/NEM resistant LPPs are expressed in the brain, yet very little is known about the role of the LPPs as regulators of LPA receptor signalling in the nervous system. Knockout studies of all the LPP subtypes have been reported [44-46]. Study with LPP1 knockout mice indicated that LPP1 plays a role in regulating the degradation of circulating LPA *in vivo* but that study failed to disrupt the LPP1 encoding gene in the brain, obscuring the function of LPP1 in the nervous system [46]. Knockout of LPP3 turned out to be embryonically lethal [45] whereas *in vitro* studies using cell lines lacking LPP3 address involvement of LPP3 in early neural development [47]. The LPPs are likely to be involved in LPA dephosphorylation in brain cryosections, as brain sections efficiently generate P_i_ from exogenous LPA largely in a NEM resistant and Mg^2+^-independent way. Propranolol and vanadate have been demonstrated to inhibit LPPs in various cell types [20,35,36,48], vanadate also in the rat brain [49]. Propranolol has been shown to act as a moderately effective inhibitor of LPPs [20] supporting our finding where the vanadate-induced response is relatively stronger when compared to the response observed with propranolol. Since propranolol and vanadate amplified LPA_1_ receptor signalling only when present in the ^35^ S]GTPγS labelling step, these drugs presumably inhibit LPPs in a reversible manner. In brain sections, LPP activity appears to locally control the lifetime of the signalling pool of LPA and LPPs must therefore reside in close proximity to the LPA_1_ receptors, as propranolol and vanadate had no effect on LPA degradation when assessed at the bulk brain level.

In functional autoradiography, AlF_x_^-^ more efficiently induced the LPA_1_ receptor-mediated signal as compared to the signals observed with vanadate or propranolol. Since AlF_x_^-^ is able to induce the LPA_1_ receptor-mediated signal when present only in the pre-incubation step, it appears to inhibit LPPs in an irreversible manner. This proposal is supported by the finding that the Al^3+^ chelator DFOM failed to reverse AlF_x_^-^ -evoked response, if added only after pretreatment of brain sections with AlF_x_^-^ (and NaF). AlF_x_^-^ is known to mimic the chemical structure of phosphate and therefore affects the activity of several phosphoryl transfer enzymes [38]. As a phosphate analog, AlF_x_^-^ might bind to the P_i_ recognizing binding pocket of the LPPs and by this mechanism lead to irreversible inhibition. All the studied inhibitors evoked ^35^ S]GTPγS binding responses that were largely restricted to the white matter areas of the brain when compared to grey matter (See Additional file 7: Inhibitor-evoked ^35^ S]GTPγS binding responses are restricted to the white matter areas of the brain) reflecting to selectivity towards the myelin-enriched LPA_1_ receptors. This also provides evidence to show, that though AlF_x_^-^ is known to act as a general activator of heterotrimeric G proteins, it seems not to induce global binding response in the grey matter areas and therefore seems not to act as a general G protein activator in functional autoradiography. It is notable that in contrast to propranolol and vanadate, when present in the latter step together with exogenous LPA, AlF_x_^-^ totally prevented the degradation of LPA at the bulk brain level, suggesting that in addition to irreversibly inhibiting LPPs, AlF_x_^-^ can inhibit other LPP-like phosphatases in a reversible manner. Based on the present findings, the major portion of brain LPA phosphatase activity appears to be attributable to the LPP-like phosphatases which in a similar manner as LPPs, are sensitive to AlF_x_^-^ but resistant to the LPP inhibitors, vanadate and propranolol.

Since there was no P_i_ generation from exogenous glycerol 3-phosphate, it seems that LPA is predominantly degraded by the LPA → MAG → G pathway in our experimental setting whereas the LPA → GP → G pathway plays a minor role. According to our findings, both phosphohydrolases (LPPs/LPP-like) and MGL and related hydrolases (ABHD6/ABHD12) seem to be active. The P_i_- and glycerol -generating enzymatic routes involved in LPA degradation are summarized in Additional file 8: Summary of enzymatic routes generating P_i_ and glycerol. Previously, NEM-insensitive LPA phosphohydrolase activity was studied in the nuclear fraction isolated from rabbit cerebral cortex [50]. This activity was found to be present also in the microsomal fraction. In the nuclear fraction, phosphohydrolase activity was found to be sensitive to NaF (50 mM) but virtually insensitive to propranolol (0.5 mM). Dephosphorylation by phosphohydrolases was found to be more active route for LPA degradation when compared to deacylation by lysophospholipases. It was also indicated that followed by dephosphorylation of LPA, monoacyl product is rapidly converted to glycerol by monoglyceride lipase. These findings support our present findings concerning active pathways involved in LPA degradation in brain as well as about the existence of LPP-like, propranolol and vanadate -insensitive, phosphohydrolase activity.

The LPP-like phosphatases remain to be characterized in future experiments. One interesting group of brain-specific membrane proteins are plasticity related genes (PRGs) that have recently been identified and were originally proposed to act as LPA phosphatases [51]. Among the family of PRGs, PRG-1 shares close homology to the LPPs, having three conserved integral domains facing the extracellular side of the plasma membrane, the feature that enables LPPs to dephosphorylate their lipid substrates. However, the catalytic residues responsible for LPP activity are not fully conserved in PRGs [51] suggesting that PRGs might not act as LPA phosphatases. Instead, PGR-1 was recently demonstrated to act at the postsynaptic side of the excitatory glutamatergic synapse where it could mediate the uptake of bioactive lipids [52]. PRG-1 was found to effectively control the levels of LPA in the synapse though its mechanism of action seems to be more transporter-like than dephosphorylating. The transporter mechanism is not expected to be active in our experimental setting and therefore we hypothesize that PRGs are not controlling LPA levels in our model.

## Conclusions

We demonstrate that the lifetime of bioactive LPA is controlled by LPPs in rat brain cryosections and that pharmacological inhibition of this LPP activity results in amplification of basal and LPA-stimulated LPA_1_ receptor activity. We conclude that LPP acts locally to control the lifetime of the signalling pool of LPA in the vicinity of LPA_1_ receptors whereas the majority of brain LPA phosphatase activity is attributable to additional LPP-like enzymatic activity. Functional autoradiography represents a valuable tool for studies into LPA degradation by LPPs and LPP-like enzymatic activity. Compounds affecting LPA degradation could prove to be attractive targets for drug development, since altered LPA levels are associated with common human diseases, e.g. several forms of cancer. The approach described in this paper may also prove useful for further studies elucidating enzymatic pathways regulating the lifetime of still uncharacterized endogenous signalling lipids.

## Methods

### Chemicals

Propranolol was purchased from Biomol (Plymouth Meeting, PA, USA). AlCl_3_ and NaF were from Merck (Darmstat, Germany). 1-oleoyl-2-methyl-*sn*-glycero-3-phosphothionate ((2S)-OMPT), 1-palmitoyl-2-hydroxy-*sn*-glycero-3-phosphate (16:0 LPA), 1-heptadecanoyl-2-hydroxy-*sn*-glycero-3-phosphate (17:0 LPA), 1-stearoyl-2-hydroxy-*sn*-glycero-3-phosphate (18:0 LPA), 1-arachidonoyl-2-hydroxy-*sn*-glycero-3-phosphate (20:4 LPA) were from Avanti Polar Lipids (Alabaster, AL, USA). 1-oleyl-2-hydroxy-*sn*-glycero-3-phosphate (18:1 LPA), sodium orthovanadate (Na_3_VO_4_), nadolol, bovine serum albumin (BSA, fatty acid free), dithiothreitol (DTT), 8-cyclopentyl-1,3-dipropylxanthine (DPCPX), guanosine diphosphate (GDP), 3-(4-[4-([1-(2 chlorophenyl)ethoxy] carbonyl amino)-3-methyl-5-isoxazolyl]benzylsulfanyl) propanoic acid (Ki16425), GTPγS, N-ethylmaleimide (NEM), glycerol 3-phosphate, and deferoxamine mesylate (DFOM) were from Sigma (St Louis, MO, USA). Methylarachidonoylfluorophosphonate (MAFP) and compound JZL184 were from Cayman Chemical (Ann Arbor, MI, USA). [^35^ S]GTPγS (initial specific activity 1250 Ci/mmol) was purchased from NEN Life Science Products Inc. (Boston, MA, USA). All other chemicals were of the finest purity available.

### Animals

Experiments were performed using 4-week old male Wistar rats obtained from the National Laboratory Animal Centre, University of Eastern Finland, Kuopio, Finland. Approval for the harvesting of animal tissue was applied, registered and obtained from the local welfare officer of the University of Eastern Finland. The experiments did not involve any *in vivo* treatment. The animals were housed in groups of five to ten individuals per cage under standard laboratory conditions (12–12 h light–dark cycle, food and water *ad libitum*, 60% relative humidity). The rats were decapitated 8–9 h after lights on, and within the next 5 min, the whole brain was dissected out, dipped briefly in isopentane (chilled on dry ice) and stored at −80 °C. Horizontal, coronal or sagittal brain sections (20 μm thick) were cut at −19 °C to −21 °C using a Leica cryostat, thaw-mounted onto Superfrost®Plus slides (Menzel-Gläser, Germany), dried for 1–4 h at room temperature under a constant stream of air and stored thereafter at −80 °C.

### [^35^ S]GTPγS autoradiography

^35^ S]GTPγS autoradiography was performed as previously described [26,28,34]. Briefly, the brain sections were processed in three sequential steps consisting of pre-incubation for 20/40 min (step 1), GDP-loading for 50/60 min (step 2), and ^35^ S]GTPγS labelling for 90 min (step 3). For some treatments, a brief additional incubation (10 min) was performed prior to step 1, as detailed in Results. All the steps were performed at 20 °C using Tris-based buffer (50 mM Tris–HCl, pH 7.4, 1 mM EDTA, 100 mM NaCl, 5 mM MgCl_2_). Steps 2 and 3 included 1 μM DPCPX (to block the tonic adenosine A_1_ receptor signal) and 2 mM GDP and step 3 additionally included 1 mM DTT and 40–250 pM ^35^ S]GTPγS. Chemicals being investigated (LPA species used was 18:1 LPA) and 0.1% BSA (fatty acid free) were included in the assay during steps 1,2 or 3 as described in Results. In addition, some slides in each experiment were incubated in the presence of 10 μM GTPγS to determine non-specific binding. After the 90 min autoradiography step, the slides were rinsed twice (5 min each time) in ice-cold washing buffer (50 mM Tris–HCl, pH 7.4 and 5 mM MgCl_2_), dipped for 30 s in ice-cold deionized water, and air-dried. The slides were arranged into a cassette together with ^14^ C] standard (Amersham, Little Chalfont, Bucks, UK) and exposed against a radiosensitive film (BioMax MR™, Kodak Scientific Imaging Film) for 2–5 days. After exposure, the films were developed for 3–4 min at 4 °C with Kodak D-19 developer.

### [^35^ S]GTPγS membrane binding assay

A previously described method was utilized for preparation of membranes [39]. Briefly, eight cerebella were homogenized in nine volumes of ice-cold 0.32 M sucrose. The homogenate was centrifuged at 1000 × g for 10 min at 4 °C and the resulting supernatant was centrifuged at 100 000 × g for 30 min at 4 °C. The high speed centrifugation was repeated twice, resuspending the pellet in ice-cold deionised water. After the final centrifugation, the membranes were suspended in Tris–HCl (50 mM, pH 7.4) supplemented with EDTA (1 mM) and stored thereafter at −80 °C.

The ^35^ S]GTPγS membrane binding assay was performed as previously described [28,34], with minor modifications. Briefly, the final incubation volume (400 μl) contained 5 μg membrane protein in the incubation buffer (50 mM Tris–HCl, pH 7.4, 1 mM EDTA, 100 mM NaCl, 5 mM MgCl_2,_ 10 μM GDP, 1 mM DTT, 0.5% BSA, and ~ 150 pM ^35^ S]GTPγS) plus the chemicals of interest. The incubation buffer was supplemented with 1 μM DPCPX to suppress basal ^35^ S]GTPγS binding due to endogenous adenosine. The membranes were pre-incubated for 30 min prior to conducting the ^35^ S]GTPγS binding step. The incubations were stopped after 90 min by the addition of 4 ml ice-cold washing buffer (50 mM Tris–HCl, pH 7.4 and 5 mM MgCl2), followed by filtration through glass fibre filters (Whatman GF/B) and by two additional washes with the buffer. The filters were transferred into scintillation vials along with HiSafe3 scintillation liquid (Wallac, Turku, Finland). After vertical shaking for 15 min to extract radioactivity trapped in filters, the tubes were counted the next day with Wallac LKB 1213 Rackbeta, Wallac, Turku, Finland.

### Determination of P_i_ and glycerol

To estimate LPA-degrading phosphatase activity under the assay conditions mimicking functional autoradiography, triplicate slides with two or three horizontal brain sections underwent the autoradiography mimicking protocol (except that DPCPX, GDP and radioligand were omitted). The experiments with Na_3_VO_4_, propranolol, and nadolol (as a negative control for propranolol) were performed with and without NEM pretreatment. The protocol consisted of 10 min pre-incubation in the assay buffer, 30 min incubation in the assay buffer with or without 5 mM NEM, then a washing step with assay buffer, and finally 90 min incubation in the presence of chemicals in interest as well as 0.1% BSA and 1 mM DTT. In NEM-treated sections, Mg^2+^ was omitted from the assay buffer. In experiments with NaF and AlF_x_^-^, the protocol consisted of two sequential 40 min incubations (steps 1 and 2) and finally 90 min incubation (step 3) in the presence of the chemicals of interest as well as 0.1% BSA and 1 mM DTT. LPA species used was 18:1 LPA. In all experiments, after the final 90 min incubation step, the postincubation buffer was collected quantitatively and the P_i_ content was determined in duplicate using the Fiske-Subbarow method, as described in Esmann (1988) [53] after TCA-precipitation of BSA which interfered with the P_i_ determinations. Absorbances (λ 700 nm) were read with a Tecan Spectrafluor microplate reader.

To clarify the enzymatic routes responsible for LPA degradation in our experimental setting, P_i_ formation was determined using enzyme-coupling fluorescent method [54]. The glycerol content was determined using a coupled enzyme reaction (Free Glycerol Reagent, Sigma, Cat.# F6428) with the exception that H_2_O_2_ production was coupled to peroxidase-dependent formation of the fluorescent dye resorufin. Briefly, rat cerebellar membranes (1 μg/well of 96-well plate), prepared as described in [39], were pretreated with the serine hydrolase inhibitors MAFP (1 μM) or JZL184 (100 μM) for 30 min and then incubated with or without 18:1 LPA, GP, or (2 S)-OMPT (10 μM final concentration). The fluorescence (λ_ex_ 530 nm, λ_em_ 590 nm) was monitored kinetically for 90 min at 10 min intervals using Tecan Infinite M200 fluorometer.

### Extraction of LPA for mass spectrometric determination

Slides with two horizontal brain sections were incubated for 40 min in the presence of AlF_x_^-^ (NaF 10 mM + AlCl_3_ 50 μM) mimicking the ^35^ S]GTPγS autoradiography. The control slides were incubated similarly in the assay buffer. After 40 min incubation, slides were rinsed twice (2 min each time) in ice-cold washing buffer, dipped for 30s in ice-cold deionized water and air-dried. The modified extraction method of Bligh and Dyer [55] was applied for the isolation of LPA from the tissue matrix. One sample consisted of pooled tissue obtained from four slides. The brain tissue was scraped manually from the slides with a spatula using the mixture of 50 mM Tris–HCl, pH 7.40 and methanol with a ratio of 1:4 (v/v); this mixture also included an internal standard (17:0 LPA) used in the quantification. The tissue was transferred to a screw-capped Pyrex® borosilicate glass test tube. The mixture of 50 mM Tris–HCl, pH 7.40 and methanol (1:4, v/v) was added to the test tube to bring the volume up to 200 μl. Chloroform was added to yield a water/methanol/chloroform ratio of 1:4:2 (v/v/v) and the samples were shaken for 1 h with a vertical shaker (Heidolph Multi Reax, Heidolph Instruments GmbH & Co, Schwabach, Germany). To achieve the phase separation, 80 μl of chloroform and 80 μl of water were added. After vortexing for 1 min, the samples were centrifuged at 1800 x *g* for 15 min at room temperature. The upper aqueous layer was transferred to an HPLC sample vial.

### Liquid chromatography/tandem mass spectrometry (LC/MS/MS)

The method for LC/MS/MS determination of LPAs with varying acyl chains has been previously described [9]. The HPLC system comprised of an Agilent 1200 Series Rapid Resolution LC System (Agilent Technologies, Waldbronn, Germany) consisting of a solvent micro vacuum degasser, a binary pump, a thermostatted column compartment SL, and an autosampler SL. Ten microliter of sample solution were injected onto a reversed phase HPLC column (XBridge™ C8 2.1x50 mm, 2.5 μm) (Waters, Ireland) using gradient elution with 50 μM ammonium acetate + 1% triethylamine (TEA) (A) and 1% TEA in 90% methanol (B) as follows: 0–6.0 min 20% B → 90% B, 6.0-10.0 min 90% B, 10.0-10.1 min 90% B → 20% B, 10.0-15.0 min 20% B. The flow rate was 0.3 ml/min, column temperature was maintained at 40 °C and the autosampler tray temperature was set to 10 °C.

The mass spectrometric analysis was carried out with an Agilent 6410 Triple Quadrupole LC/MS equipped with an electrospray ionization source (Agilent Technologies, Palo Alto, CA, USA). The following ionization conditions were used: ESI negative ion mode, drying gas (nitrogen) temperature 300 °C, drying gas flow rate 8 l/min, nebulizer pressure 40 psi and capillary voltage 4000 V. Analyte detection was performed using multiple reaction monitoring (MRM) with the following transitions: *m/z* 409 → 153 for 16:0 LPA, *m/z* 437 → 153 for 18:0 LPA, *m/z* 435 → 153 for 18:1 LPA, *m/z* 457 → 153 for 20:4 LPA, and *m/z* 423 → 153 for 17:0 LPA. The fragmentor voltage was 160 V and collision energy 20 V except 23 V for 17:0 LPA. Data were acquired by Agilent MassHunter Workstation Acquisition software (Agilent Technologies, Data Acquisition for Triple Quad., version B.01.03).

### Data analysis

Autoradiography films were digitized using a HP scanjet 7400c scanner. For the quantitative data, optical densities on the autoradiograms were measured using ImageJ, a freely available java-based image analysis software system developed in the National Institutes of Health, USA (http://rsb.info.nih.gov/ij/). Optical densities were converted to nCi/g using nonlinear transformation built by the greyscale values of [^14^ C] standards. In LC/MS/MS experiments, an internal standard (17:0 LPA) was used for quantification, and peak area ratios of the analyte to the IS were calculated as a function of the concentration ratios of the analyte to the internal standard using Agilent MassHunter software (Quantitative Analysis Version B.01.03). The protein content of brain sections was determined by the Pierce BCA Protein Assay Kit with BSA as the standard. The statistical differences were determined either using one-way ANOVA with Tukey’s multiple comparison *post hoc* test or *t* test (LC/MS/MS experiments) with ***p < 0.001, **p < 0.01, or *p < 0.05 considered as statistically significant. All statistical data analyses were conducted using GraphPad Prism for Windows.

## Abbreviations

2-AG, 2-arachidonoylglycerol; AlFx-, Aluminium fluoride; BSA, Bovine serum albumin; DFOM, Deferoxamine mesylate; GP, Glycerol phosphate; GPCR, G protein-coupled receptor; LC/MS/MS, Liquid chromatography/tandem mass spectrometry; LPA, Lysophosphatidic acid; LPP, Lipid phosphate phosphatase; MAG, Monoacylglycerol; MGL, Monoglyceride lipase; NaF, Sodium fluoride; Na3VO4, Sodium orthovanadate; NEM, N-ethylmaleimide; PA, Phosphatidic acid; Pi, Inorganic phosphate; S1P, Sphingosine 1-phosphate.

## Competing interests

The authors declare that they have no competing interests.

## Authors’ contributions

NA carried out the LC/MS/MS determinations, supervised and partly conducted the autoradiography experiments and quantification of the results, and drafted the manuscript. ML provided the facilities and carried out the design of the LC/MS/MS determinations. M.Sc. student KV for the most part carried out the autoradiography experiments and quantification of the results, and participated in the P_i_ determinations. Erasmus exchange student GAG for the most part carried out the P_i_ determinations. JTL initiated the study, carried out its design and coordination and helped to draft the manuscript as a senior author. All authors read and approved the final manuscript.

## Supplementary Material

Additional file 1 **The maximal effective concentrations for Na**_**3**_**VO**_**4**_**and propranolol.** (Autoradiography image) (PDF 290 kb) Click here for file

Additional file 2 **Propranolol, but not nadolol, induces LPA mimicking binding response.** (Autoradiography image) (PDF 164 kb) Click here for file

Additional file 3 **Propranolol and vanadate do not activate LPA**_**1**_**receptors.** (Graph) (PDF 81 kb) Click here for file

Additional file 4 **Comparison of the [**^**35**^** S]GTPγS binding responses between LPA and (2 S)-OMPT.** (Autoradiography image) (PDF 248 kb) Click here for file

Additional file 5 **Autotaxin is not responsible for tonic LPA**_**1**_**activity.** (Autoradiography image) (PDF 102 kb) Click here for file

Additional file 6 **Phosphate generation from exogenous LPA, PA, and S1P.** (Graph) (PDF 15 kb) Click here for file

Additional file 7 **Inhibitor-evoked [**^**35**^** S]GTPγS binding responses are restricted to the white matter areas of the brain.** (Graph) (PDF 60 kb) Click here for file

Additional file 8 **Summary of enzymatic routes generating P**_**i**_**and glycerol.** (Graph) (PDF 25 kb) Click here for file
