# Supplementary material for: Lipid phosphate phosphatase inhibitors locally amplify lysophosphatidic acid LPA1 receptor signalling in rat brain cryosections without affecting global LPA degradation
Source: BMC Pharmacol. 2012 Jun 11;12:7. doi: 10.1186/1471-2210-12-7 (PMC3418163; doi:10.1186/1471-2210-12-7)
Supplement: Additional file 1 — The maximal effective concentrations for Na3VO4and propranolol. (Autoradiography image) (PDF 290 kb) [file 1471-2210-12-7-S1.pdf]

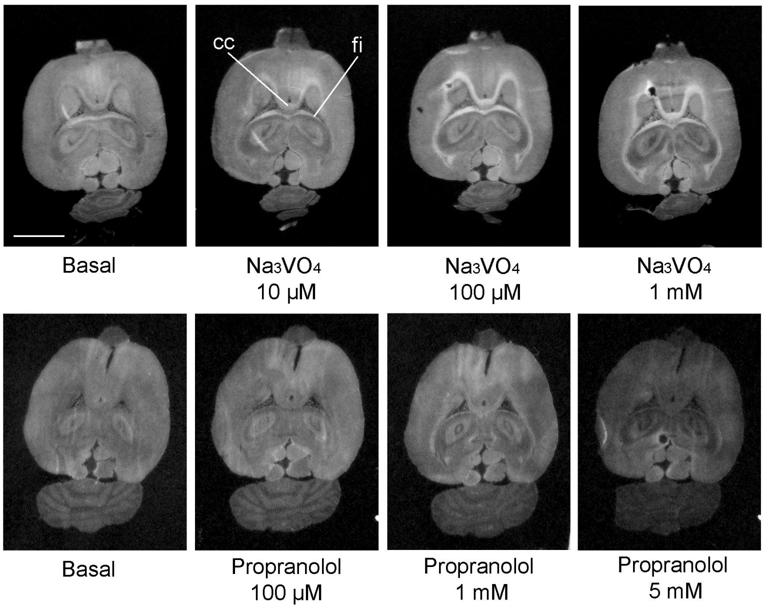

Additional file 1. The maximal effective concentrations for Na_3VO_4 and propranolol in amplifying basal LPA_1 receptor activity are 100 µM and 1 mM, respectively. Functional autoradiography using horizontal sections of 4 week-old rat brain was performed using a three-step protocol as detailed in Methods. Vanadate or propranolol were included at the indicated concentrations during the $[^{35}S]GTP\gamma S$ labelling step (step 3) which additionally contained 0.1 % BSA. Treatment with Na_3VO_4 or propranolol results in G protein activity in the LPA_1 receptor enriched white matter tracts (cc, corpus callosum; fi, fimbria of the hippocampus). A modest response is evident with 10 µM Na_3VO_4 and the maximal effective concentration is 100 µM. The maximal effective concentration of propranolol is 1 mM whereas a 10-fold lower concentration is ineffective and a 5-fold higher concentration decreases the overall binding. Scale bar = 5 mm.
