# Supplementary material for: Lipid phosphate phosphatase inhibitors locally amplify lysophosphatidic acid LPA1 receptor signalling in rat brain cryosections without affecting global LPA degradation
Source: BMC Pharmacol. 2012 Jun 11;12:7. doi: 10.1186/1471-2210-12-7 (PMC3418163; doi:10.1186/1471-2210-12-7)
Supplement: Additional file 2 — Propranolol, but not nadolol, induces LPA mimicking binding response. (Autoradiography image) (PDF 164 kb) [file 1471-2210-12-7-S2.pdf]

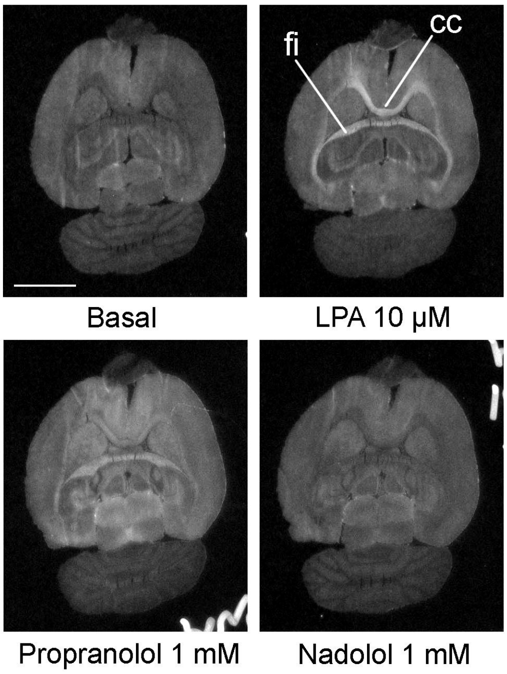

Additional file 2. Propranolol, but not nadolol, evokes LPA mimicking binding response in the white matter areas of rat brain. Horizontal brain sections were incubated using a three-step autoradiography protocol as detailed in Methods. Test chemicals were included during the [^{35}S]GTPγS labelling step (step 3) in the presence of 0.1 % BSA. Treatment with propranolol (1 mM), but not with control compound nadolol (1 mM), results in G protein activity in the LPA_1 receptor enriched white matter tracts, a response that is mimicked by exogenous LPA (10 µM) (cc, corpus callosum: fi, fimbria of the hippocampus). Scale bar = 5 mm.
