# Supplementary material for: Lipid phosphate phosphatase inhibitors locally amplify lysophosphatidic acid LPA1 receptor signalling in rat brain cryosections without affecting global LPA degradation
Source: BMC Pharmacol. 2012 Jun 11;12:7. doi: 10.1186/1471-2210-12-7 (PMC3418163; doi:10.1186/1471-2210-12-7)
Supplement: Additional file 3 — Propranolol and vanadate do not activate LPA1receptors. (Graph) (PDF 81 kb) [file 1471-2210-12-7-S3.pdf]

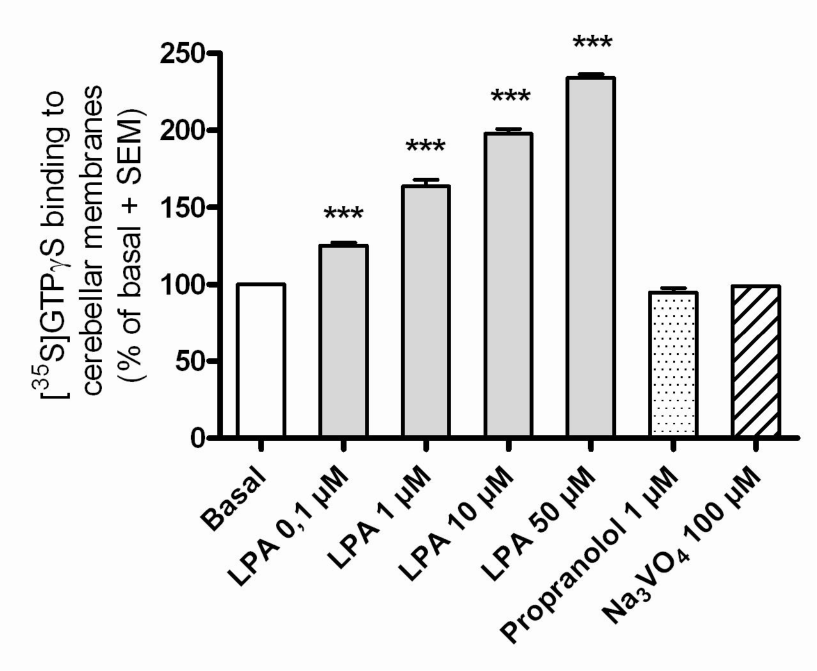

Additional file 3. Propranolol and Na_3VO_4 do not act as direct agonists for LPA_1 receptors. Classical [^{35}S]GTPγS membrane binding assay was performed as described in Methods. Propranolol and Na_3VO_4 are not able to stimulate [^{35}S]GTPγS binding to the rat cerebellar membranes, whereas exogenous LPA evokes a dose-dependent response. The data represent the percentage of bound radioactivity + SEM from three independent experiments performed in duplicate (n=3). Significance level: ***p < 0.001 compared to basal.
