# Supplementary material for: Lipid phosphate phosphatase inhibitors locally amplify lysophosphatidic acid LPA1 receptor signalling in rat brain cryosections without affecting global LPA degradation
Source: BMC Pharmacol. 2012 Jun 11;12:7. doi: 10.1186/1471-2210-12-7 (PMC3418163; doi:10.1186/1471-2210-12-7)
Supplement: Additional file 4 — Comparison of the [35 S]GTPγS binding responses between LPA and (2 S)-OMPT. (Autoradiography image) (PDF 248 kb) [file 1471-2210-12-7-S4.pdf]

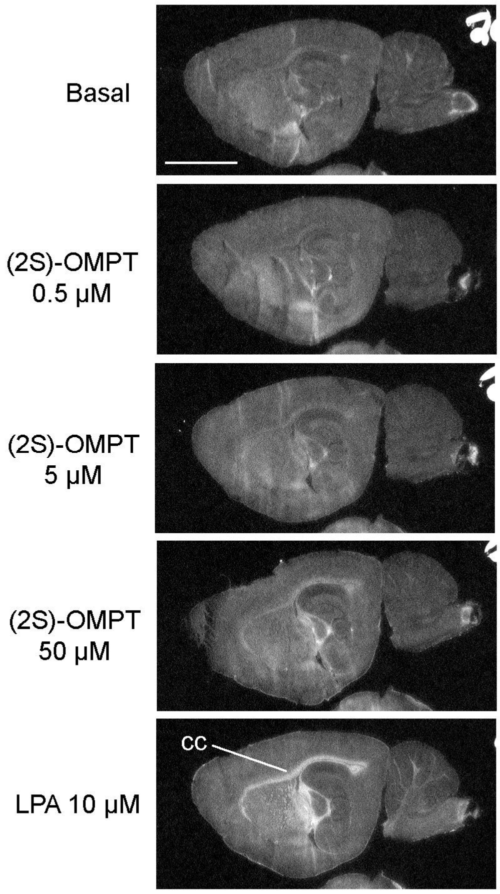

Additional file 4. In [^{35}S]GTPγS autoradiography, the LPA_3 receptor-preferring agonist (2S)-OMPT induces only a weak response as compared to the signal evoked by LPA. Functional autoradiography using sagittal sections of 4 week-old rat brain was performed using a three-step protocol as described in Methods. (2S)-OMPT or LPA were included during the [^{35}S]GTPγS labelling step which additionally contained 0.1 % BSA. (2S)-OMPT (0.5 µM to 50 µM) induces a weak dose-dependent signal in the white matter tracts. Even with the highest concentration (50 µM), (2S)-OMPT-evoked response was considerably weaker than that evoked by LPA (10 µM) (cc, corpus callosum). Scale bar = 5 mm.
