# Supplementary material for: Lipid phosphate phosphatase inhibitors locally amplify lysophosphatidic acid LPA1 receptor signalling in rat brain cryosections without affecting global LPA degradation
Source: BMC Pharmacol. 2012 Jun 11;12:7. doi: 10.1186/1471-2210-12-7 (PMC3418163; doi:10.1186/1471-2210-12-7)
Supplement: Additional file 5 — Autotaxin is not responsible for tonic LPA1activity. (Autoradiography image) (PDF 102 kb) [file 1471-2210-12-7-S5.pdf]

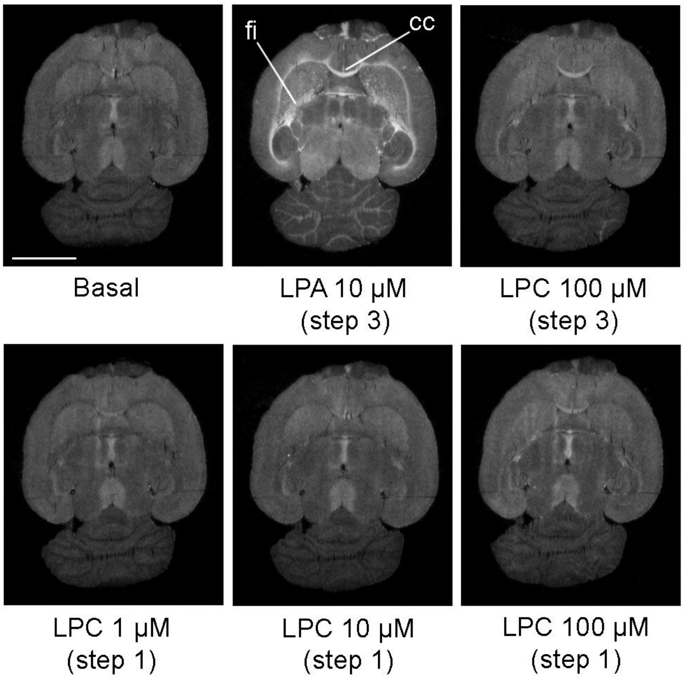

Additional file 5. Evidence indicating that tonic LPA_1 receptor activity under basal conditions of [^{35}S]GTPγS autoradiography is not due to autotaxin activity. Functional autoradiography using horizontal sections of 4 week-old rat brain was performed using a three-step protocol as detailed in Methods. Exogenous LPA (10 µM) was added during step 3 which additionally contained 0.1 % BSA. Incubation of brain sections with the autotaxin substrate lysophosphatidylcholine (LPC) (1 µM to 100 µM, step 1 or 3 in the presence of 0.1 % BSA) does not affect tonic LPA_1 receptor activity (cc, corpus callosum; fi, fimbria of the hippocampus). Scale bar = 5 mm.
