# Supplementary material for: Lipid phosphate phosphatase inhibitors locally amplify lysophosphatidic acid LPA1 receptor signalling in rat brain cryosections without affecting global LPA degradation
Source: BMC Pharmacol. 2012 Jun 11;12:7. doi: 10.1186/1471-2210-12-7 (PMC3418163; doi:10.1186/1471-2210-12-7)
Supplement: Additional file 6 — Phosphate generation from exogenous LPA, PA, and S1P. (Graph) (PDF 15 kb) [file 1471-2210-12-7-S6.pdf]

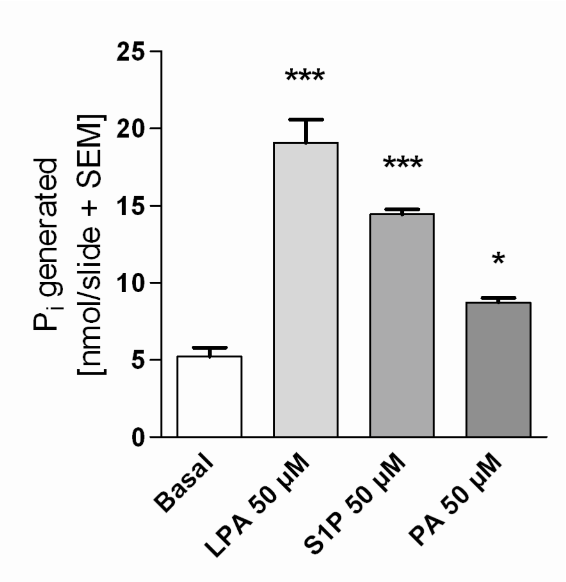

Additional file 6. Incubation of rat brain sections with exogenous LPA, PA and S1P results in P_i generation indicating that lysophospholipid-degrading phosphatases are active in the assay conditions used for functional autoradiography. Slides with two horizontal brain sections underwent the autoradiography mimicking incubation protocol, as detailed in Methods. Following 90 min incubation in the presence of 0.1 % BSA together with the indicated compounds, the assay buffer was quantitatively collected and the P_i content was determined as described in Methods. Treatment of brain sections with exogenous LPA (50 µM), S1P (50 µM), and PA (50 µM) generates significant amounts of P_i when compared to the basal condition. Brain sections dephosphorylate the lysophospholipids with the relative preference order LPA > S1P > PA. The data are expressed as nmol P_i generated per slide (mean + SEM) performed in triplicate (n=3). Significance level: ***$p < 0.001$ and *$p < 0.05$ compared to basal.
