# Supplementary material for: Lipid phosphate phosphatase inhibitors locally amplify lysophosphatidic acid LPA1 receptor signalling in rat brain cryosections without affecting global LPA degradation
Source: BMC Pharmacol. 2012 Jun 11;12:7. doi: 10.1186/1471-2210-12-7 (PMC3418163; doi:10.1186/1471-2210-12-7)
Supplement: Additional file 7 — Inhibitor-evoked [35 S]GTPγS binding responses are restricted to the white matter areas of the brain. (Graph) (PDF 60 kb) [file 1471-2210-12-7-S7.pdf]

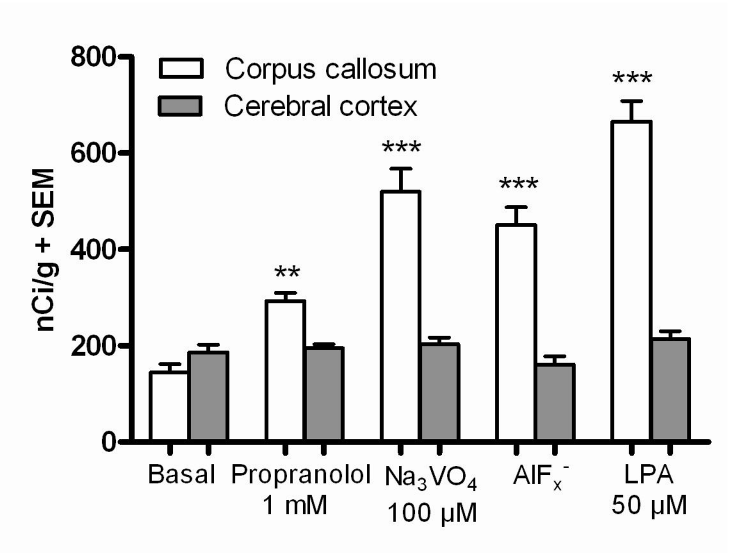

Additional file 7. Inhibitor-evoked [^{35}S]GTPγS binding responses are restricted to white matter areas of rat brain. Coronal brain sections were incubated using a three-step autoradiography protocol as detailed in Methods. AlF_x^- (NaF 10 mM + $AlCl_3$ 50 µM) was included during the 40 min preincubation step (step 1) and other test chemicals during the [^{35}S]GTPγS labelling step (step 3). Step 3 additionally contained 0.1 % BSA. Autoradiography films were digitized and [^{35}S]GTPγS binding was quantified from the corpus callosum (representing white matter) or from cerebral cortex (representing grey matter), as described in Methods. Quantitative data are calculated as nCi/g equivalents with non-specific binding subtracted from total binding. The data are expressed as mean + SEM from six individual animals (n=6). Significance level: ***$p < 0.001$ and **$p < 0.01$ compared to basal in each brain region.
