# Supplementary material for: Lipid phosphate phosphatase inhibitors locally amplify lysophosphatidic acid LPA1 receptor signalling in rat brain cryosections without affecting global LPA degradation
Source: BMC Pharmacol. 2012 Jun 11;12:7. doi: 10.1186/1471-2210-12-7 (PMC3418163; doi:10.1186/1471-2210-12-7)
Supplement: Additional file 8 — Summary of enzymatic routes generating Piand glycerol. (Graph) (PDF 25 kb) [file 1471-2210-12-7-S8.pdf]

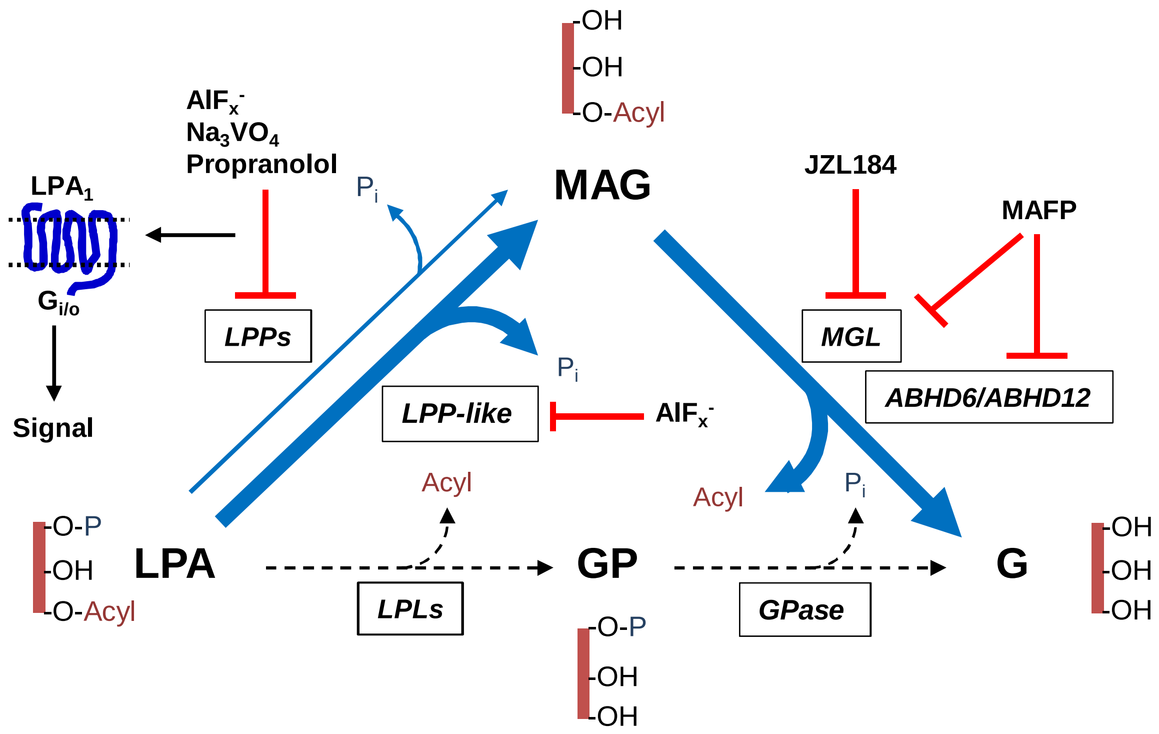

Additional file 8. Summary of the P_i- and glycerol-generating enzymatic pathways potentially involved in brain tissue-dependent LPA degradation. In the LPA → MAG → G pathway, lipid phosphate phosphatases (LPPs) and LPP-like phosphatases dephosphorylate LPA resulting in the generation of equimolar amounts of monoacylglycerol (MAG) and P_i. MAG is further hydrolyzed by monoglyceride lipase (MGL), resulting in cleavage of the acyl moiety and the formation of glycerol (G). Aluminium fluoride (AlF$_x^-$) irreversibly and sodium orthovanadate (Na_3VO_4) and propranolol reversibly inhibit LPPs guarding the signalling-pool of LPA. In addition, AlF$_x^-$ reversibly and comprehensively inhibits the LPP-like phosphatases that in brain sections degrade the bulk of LPA. Compound JZL184 selectively inhibits MGL whereas methylarachidonoylfluorophosphonate (MAFP) inhibits both MGL and α/β-hydrolase domain containing proteins ABHD6 and ABHD12. In the LPA → GP → G pathway, lysophospholipases (LPLs) catalyze the deacylation of LPA with the concomitant formation of glycerophosphate (GP). GP can be further metabolized by GP phosphatase (GPase) activity thereby generating equimolar amounts of P_i and G. We found that this pathway is not involved in LPA degradation. The width of the arrows indicates the relative activity of the pathway in the present experimental setting. According to the present study, the LPA → MAG → G pathway is predominantly responsible for LPA degradation in rat brain whereas the LPA → GP → G pathway plays a minor role. The majority of brain LPA phosphatase activity is attributed to LPP-like enzymatic activity. The blunt arrows indicate the enzyme inhibition.
